# Change of joint-line convergence angle should be considered for accurate alignment correction in high tibial osteotomy

**DOI:** 10.1186/s43019-020-00076-x

**Published:** 2021-01-11

**Authors:** Young Gon Na, Beom Koo Lee, Ji Uk Choi, Byung Hoon Lee, Jae Ang Sim

**Affiliations:** 1 Department of Orthopedic Surgery, CM Hospital, Yeongdeungpo-ro 36-gil, Yeongdeungpo-gu, 07301 South Korea; 2 Department of Orthopedic Surgery, Gachon University Gil Medical Center, 21, Namdong-daero 774 beon-gil, Namdong-gu, Incheon, 21565 South Korea

**Keywords:** Knee, Osteoarthritis, High tibial osteotomy, Alignment, Joint-line convergence angle

## Abstract

**Background:**

The alignment correction after high tibial osteotomy (HTO) is made both by bony correction and soft-tissue correction around the knee. Change of the joint-line convergence angle (JLCA) represents the soft-tissue correction after HTO, which is the angle made by a tangential line between the femoral condyles and the tibial plateau. We described the patterns of JLCA change and related factors after HTO and investigated the appropriate preoperative planning method.

**Methods:**

Eighty patients who underwent HTO between 2013 and 2016 were included for this retrospective study. Standing, whole-limb radiograph, supine knee anteroposterior (AP) and lateral were measured on the preoperative and postoperative radiographs. The patterns of JLCA changes and related factors were analyzed.

**Results:**

JLCA decreased by a mean of 0.9° ± 1.2° (*P* < 0.001) after HTO. Sixteen patients (20%, group II) showed a greater JLCA decrease ≥ 2°, while 64 (80%, group I) patients remained in a narrow range of JLCA change < 2°. Group II showed more varus deformity (varus 8.1° vs. varus 4.7° in the mechanical femorotibial angle, *P* < 0.001), greater JLCA on standing (4.9° vs. 2.1°, *P* < 0.001), and the difference of JLCA in the standing and supine positions (2.8° vs. 0.7°, *P* < 0.001) preoperatively compared to group I. The risk of a greater JLCA decrease ≥ 2° was associated with greater preoperative JLCA in the standing position and the difference between the JLCA in the standing and supine positions. Postoperative JLCA correlated better with preoperative JLCA in the supine position than those in the standing position. A preoperative JLCA ≥ 4° or the difference of preoperative JLCA in the standing and supine positions ≥ 1.7° was the cut-off value to predict a large JLCA decrease ≥ 2° after HTO in the receiver operating characteristic (ROC) curve analysis.

**Conclusions:**

Surgeons should consider the effect of the JLCA change during the preoperative planning and intraoperative procedure to avoid unintended overcorrection.

## Background

High tibial osteotomy (HTO) is a good surgical option for younger, more active patients with medial compartment osteoarthritis (OA) with varus deformity, as it preserves the native knee joint [1–4]. HTO is an alignment correction procedure, which enables the load of the body transfer to the intact lateral compartment, rather than the osteoarthritic medial compartment [1–4]. Accurate alignment correction achievement is the key factor of successful HTO outcome; precise preoperative planning and a meticulous intraoperative procedure is required [5–7].

Many preoperative planning methods are described in the literature [8–12], but the concept of calculating the correction angle of the proximal tibia to achieve intended lower-limb alignment after HTO, i.e., “bony correction,” is similar among them. The required bony correction is mathematically calculable and predictable. However, the change of the limb alignment is also caused by both the bony correction and the “soft-tissue correction” [13–15]. The change of the joint-line convergence angle (JLCA), defined as the angle made by a tangential line between the femoral condyles and the tibial plateau, can represent the soft-tissue correction after HTO [13, 16]. As the joint-loading axis is moved from the medial compartment to the lateral compartment after HTO, the lateral joint opening of the varus osteoarthritic knee joint can close spontaneously, and the limb alignment can be corrected more than the bony correction [13, 17, 18]. JLCA change is a risk factor for the discrepancy of limb alignment both checked intraoperatively using fluoroscopy and measured on the postoperative, standing, whole-leg radiograph [19, 20]. This phenomenon can also cause unintended overcorrection of the limb alignment [13, 15]. Several studies have reported methods to consider the change of the JLCA [8, 14, 21, 22], Postoperative JLCA change is not easy to be expected and difficult to calcuate the estimated amount during the preoperative planning [15].

Therefore, we investigated the pattern of the JLCA change and its related factors after HTO and assessed preoperative planning methods to consider the JLCA change after HTO.

## Methods

### Study design and patient selection

This retrospective cohort study was approved by the Ethical Board of our institution (No. GAIRB2017–349). Eligibility for this study was evaluated for 92 consecutive patients who underwent medial open-wedge HTO for medial compartment knee OA at our hospital between 2013 and 2016. HTO was considered in patients experiencing severe knee pain which was not sufficiently controlled by more than 3 months of conservative management, owing to the medial compartment knee OA with varus limb alignment, with an intact lateral compartment. Twelve patients were excluded for the following reasons: appropriate radiograph unavailable (*n* = 2), revision HTO (*n* = 2), deformity after fracture (*n* = 2), postoperative surgical site infection (*n* = 1), lateral hinge fracture resulting in fixation loss (*n* = 1), concurrent posterolateral rotatory instability (*n* = 2), concurrent femoral lengthening procedure (*n* = 1), and less than 3 months of postoperative records available (*n* = 1). Of 92 patients, only 80 (86%) were included in this study. There were 67 women and 13 men with a mean age of 57.4 years (standard deviation (SD) = 7.4, range 31–80) and a mean body mass index (BMI) of 26.3 kg/m^2^ (SD = 3.7, range 20.0–36.1). The operation was conducted by two experienced surgeons (47 and 33 cases, respectively), and the laterality was similar between both sides (right: left = 38: 42).

### Surgical technique and rehabilitation

The surgical technique of the two operators was the same between surgeons as described in previously reported studies [14, 23]. Preoperative planning used double-limb, standing, whole-leg radiography to determine the amount of the corrective angle required. The determined angle was interpreted into the correction width of the amount of the opening gap of the osteotomy site using the actual-size printed radiograph, followed the “weight-bearing scanogram technique,” reported by Lee et al. [9] The correction target was to make the mechanical axis within 10–20% lateral to the center of the knee joint [14]. Before the final fixation, we checked the corrected limb alignment again using fluoroscopy and the cable method with the axial compression force applied to the heel to simulate the weight-bearing status postoperatively. The fixation was performed using either two locking plates (TomoFix® (DePuy Synthesis, West Chester, PA, USA) and OhtoFix® (Ohtomedical Co. Ltd., Goyang, Korea)). The range of motion was allowed immediately after surgery and full weight-bearing was permitted 8 weeks post surgery.

### Radiographic measurements

Preoperative radiographic evaluations were performed using double-limb, standing, full-length, lower-limb radiography, and anteroposterior (AP) and lateral radiographs of the knee in the supine position. We measured the mechanical femorotibial angle (mFTA, a negative value was designated to the knee in varus alignment.), the mechanical medial proximal tibial angle (mMPTA), the mechanical lateral distal femoral angle (mLDFA) JLCA, following Paley et al. [24]. The JLCA was defined as the angle made by the two tangential lines between the medial and lateral femoral condyles and the tibial plateau, for which the lateral opening was designated the positive value [16, 25]. The mFTA, mMPTA and mLDFA were measured on the double-limb, standing, whole-leg radiograph only, while JLCA was evaluated both on the double-limb, standing, whole-leg radiograph and on the supine knee AP radiograph. The posterior tibial slope was measured on the lateral view of the knee, which was defined as the angle formed between the tangential line between the medial tibial plateau and the perpendicular line of the anatomical axis of the proximal tibia.

The postoperative coronal radiographic parameters (mFTA, mMPTA, JLCA) were measured only on the double-limb, standing, whole-leg radiograph, selected from those obtained at least 3 months after surgery. JLCA change was defined as the difference of the postoperative JLCA and the preoperative JLCA, measured on the double-limb, standing, whole-leg radiograph. A negative value indicates that the JLCA is decreased after HTO. The postoperative tibial slope was measured on the lateral radiograph of the knee. The coronal alignment outlier was defined as the mFTA above the acceptable range of 0–6° [26].

All radiographs were taken with the patella facing forward and radiographs with significant rotation were not used in the radiographic measurement. All radiographic images were digitally acquired using a picture archiving communication system (PACS). Radiographic measurements were conducted using PACS software (Infinite, Seoul, Korea). The minimal detectable difference of this software was 0.1° in angle and 0.1 mm in length measurements. Two orthopedic surgeons performed radiographic measurement twice with a 2-week interval to evaluate the intra-observer and inter-observer reliabilities. The intra-and inter-observer reliabilities of the radiographic measurements were evaluated using intra-class correlation coefficients (ICCs). The ICCs of all radiographic measurement were more than 0.8 in all parameters. In the final analyses, the mean values of the measurements taken by two investigators were used.

### Statistical analysis

Statistical analyses were performed using SPSS (IBM SPSS Statistics for Windows, Version 18.0. IBM Corp., Armonk, NY, USA). To demonstrate the changing pattern of the JLCA after HTO, the amount of JLCA change was evaluated. We regarded a JLCA change of ≥ 2° as a clinically significant change, so the patients were divided into two groups: JLCA change < 2° vs. JLCA decrease ≥ 2° change. Demographic factors and preoperative and postoperative operative radiographic parameters (mFTA, mMPTA, mLDFA, JLCA) were compared between groups. Statistical significance was tested using the chi-square test or Fisher’s exact test for the categorical variables, and Student’s *t* test for the continuous variables. The relation between the amount of JLCA change after surgery with the demographic factors or radiographic parameters was estimated with Pearson’s correlation coefficient. Factors associated with a JLCA change ≥ 2° were investigated using binary logistic regression among the demographic factors and radiographic parameters. To determine whether the radiographic parameters predict the JLCA decrease ≥ 2°, receiver operating characteristic (ROC) curve analysis was performed. To develop the prediction model of postoperative JLCA on standing using several preoperative radiographic parameters, multivariable linear regression was used with the stepwise mode. The association of preoperative JLCA measurements on the different type of radiographs (standing and supine) and postoperative JLCA in the standing position were evaluated using a paired *t* test and Pearson’s correlation analysis. Null hypotheses of no difference were rejected if the *P* values were < 0.05. We estimated the sample size required to detect the mean difference of the JLCA between preoperative and postoperative values using a paired *t* test with a type I error of 0.05 and a power of 0.8, using the result of a recent previous study [17]. The a priori sample size estimation required 16 knees. Our study included 80 patients for this study, sufficient to detect the minimum meaningful difference of the JLCA change.

## Results

**Fig. 1:**
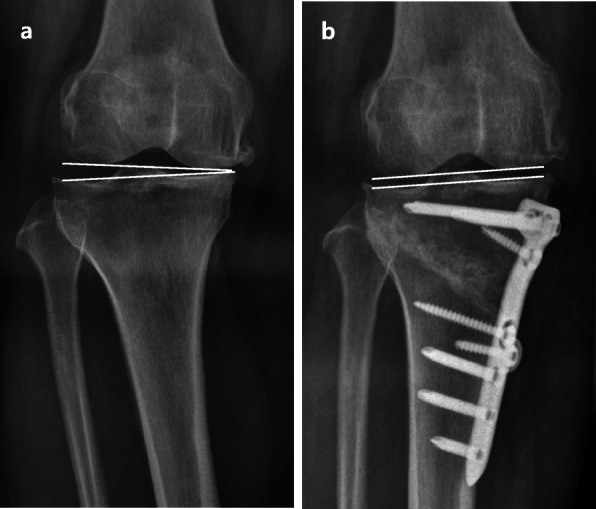
A significant change of the joint-line convergence angle before and after high tibial osteotomy. **a** preoperative, **b** postoperative

**Fig. 2:**
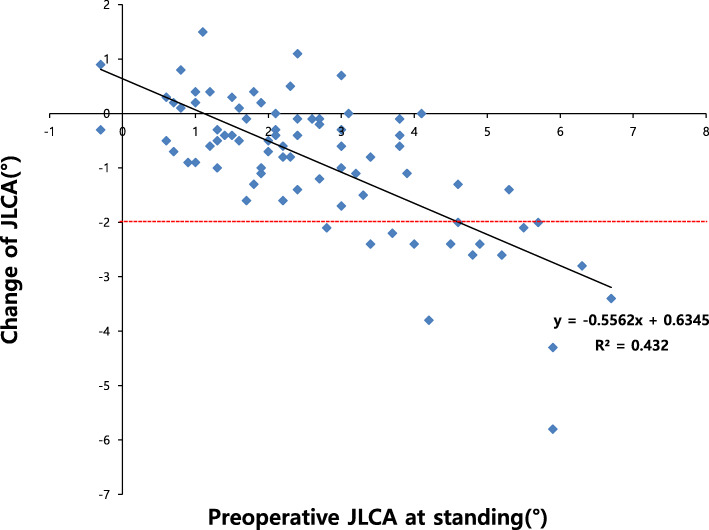
Scatter plot of the change of joint-line convergence angle (JLCA) after high tibial osteotomy according to the preoperative JLCA

JLCA decreased by a mean of 0.9° ± 1.2° (95% CI − 1.2° to − 0.6°, *P* < 0.001) after HTO. Sixteen patients (20%, group II) showed a JLCA decrease ≥ 2°, while 64 (80%, group I) patients remained in a narrow range of JLCA change < 2°. Group II showed more varus deformity (varus 8.1° vs. varus 4.7° in the mFTA, *P* < 0.001), greater JLCA on standing (4.9° vs. 2.1°, *P* < 0.001) or supine (2.1° vs. 1.4°, *P* = 0.042), and greater differences of JLCA on standing and in the supine position (2.8° vs. 0.7°, *P* < 0.001) preoperatively compared to group I (Table 1). When dividing the patients by the preoperative JLCA of 2°, 3°, and 4°, the proportion of patients with a JLCA change of ≥ 2° after HTO was significantly higher if the preoperative JLCA was greater than the cut-off values (Table 2 and Fig. 2). Among the patients with a preoperative JLCA of ≥ 4°, 81% of the patients presented a JLCA change ≥ 2° while this was only 5% in the patients with preoperative JLCA < 4° (Fig. 1).

**Table 1:** Comparison of radiographic parameters between the group with a JLCA change < 2° (group I) and the group with a JLCA decrease ≥ 2° (group II)

	JLCA change < 2° (*N* = 64)	JLCA decrease ≥ 2° (*N* = 16)	*P* value
Preoperative mFTA	− 4.7° ± 2.6°	− 9.1° ± 3.8°	**< 0.001**
Postoperative mFTA	3.4° ± 2.6°	2.6° ± 3.0°	0.301
Preoperative JLCA (standing)	2.1° ± 1.1°	4.9° ± 1.1°	**< 0.001**
Postoperative JLCA (standing)	1.7° ± 1.1°	2.1° ± 1.1°	0.262
Preoperative JLCA (supine)	1.4° ± 1.0°	2.1° ± 1.2°	**0.042**
Postoperative JLCA (supine)	1.5° ± 1.1°	1.7° ± 1.2°	0.530
Preoperative differences of JLCA on standing and when supine	0.7° ± 0.7°	2.8° ± 1.0°	**< 0.001**
Postoperative differences of JLCA on standing and when supine	0.2° ± 0.9°	0.3° ± 0.8°	0.563
Preoperative mMPTA	85.7° ± 1.9°	84.7° ± 1.7°	0.061
Postoperative mMPTA	93.2° ± 2.8°	94.5° ± 2.7	0.088
Preoperative mLDFA	88.2° ± 1.7°	89.0° ± 2.7°	0.314
Preoperative TPS	10.0° ± 2.9°	10.9° ± 3.5°	0.261
Postoperative TPS	10.5° ± 3.1°	11.8° ± 3.8°	0.179
Postoperative outlier (mFTA < 0° or > 6°)	14 (22%)	5 (31%)	0.514
Postoperative outlier (WBL < 50% or > 70%)	24 (38%)	9 (56%)	0.173

*JLCA* joint-line convergence angle, *mFTA* mechanical femorotibial angle, *mPTA* mechanical proximal tibial angle, *mLDFA* mechanical lateral distal femoral angle, *TPS* tibial posterior slope, *WBL* weight-bearing line

**Table 2:** Proportion of patients who showed decrease of JLCA on standing over 2°

Grouping by preoperative JLCA on standing	JLCA decrease ≥ 2°	*P* value
< 2° vs. ≥ 2°	0% vs. 32%	0.001
< 3°vs. ≥ 3°	2% vs*.* 48%	< 0.001
< 4° vs. ≥ 4°	5% vs. 81%	< 0.001

*JLCA* joint-line convergence angle

**Fig. 3:**
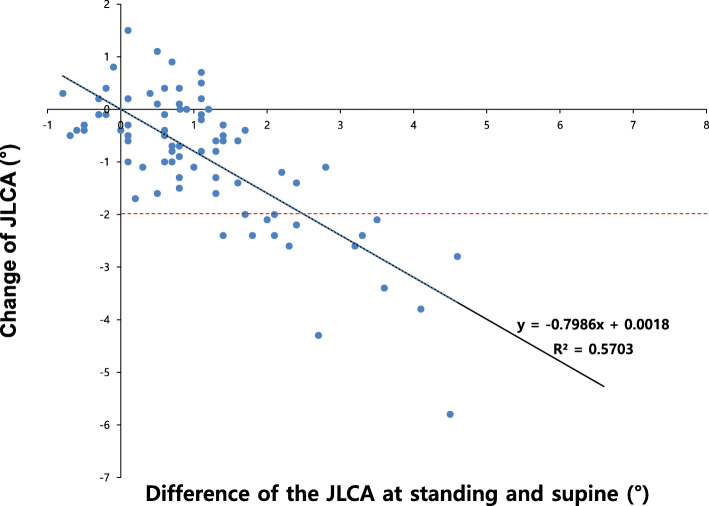
Scatter plot of the change of joint-line convergence angle (JLCA) after high tibial osteotomy according to the difference in preoperative JLCA on standing and in the supine position

**Fig. 4:**
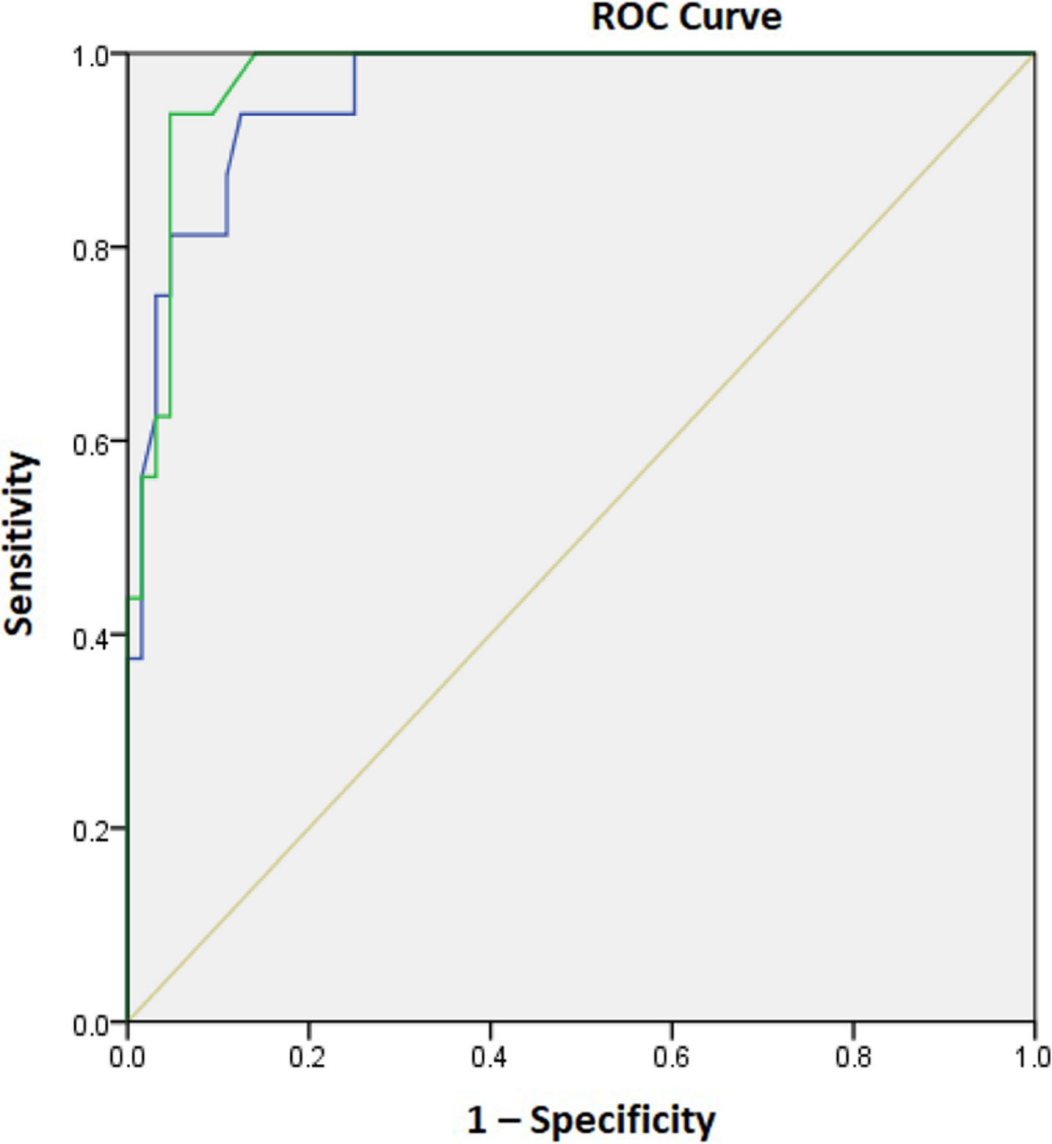
Receiver operating characteristics (ROC) curve for the joint-line convergence angle (JLCA) decrease ≥ 2° (Blue line: Preopreative JLCA at standing, Green line: Differenceof JLCA at standing and supine, Yellow line: Reference Line)

Change of JLCA was related to several radiographic parameters while demographic factors were not related (Table 3). Among the preoperative parameters, mFTA, JLCA on standing (Fig. 2) or in the supine position, the difference between the JLCA on standing and in the supine position (Fig. 3) was correlated with the amount of JLCA change: more varus limb alignment, more lateral joint opening, and greater difference of the JLCA on standing and in the supine position are correlated with a greater decrease of JLCA. Postoperative mMPTA, amount of changes of mFTA, and mMPTA were also correlated with the amount of change: greater postoperative valgus geometry of the proximal tibia, more coronal alignment correction and more tibial correction were correlated with a greater JLCA decrease. In the multivariable logistic regression model, only the preoperative JLCA on standing (odds ratio (OR) 4.429 (95% CI 1.350–14.534, *P* = 0.014) and the difference between the JLCA on standing and in the supine position (OR 11.74 (95% CI 1.770–77.886, *P* = 0.011) was associated with the risk of JLCA decrease ≥ 2°. In the ROC curve analysis, the preoperative JLCA and the difference in preoperative JLCA on standing and in the supine position were excellent predictors of JLCA decrease ≥ 2° (area under the curve of 0.959 and 0.974, respectively, both *P* values < 0.001) (Fig. 4). The preoperative JLCA ≥ 4° or the difference of preoperative JLCA on standing and in the supine position ≥ 1.7° was the cut-off value to predict a large JLCA decrease ≥ 2° after HTO in the ROC curve analysis with high sensitivity(81% and 94%, respectively) and specificity (95% and 95%, respectively).

**Table 3:** Factors affecting the amount of changes of joint-line convergence angle (JLCA)

Parameters	Correlation coefficient with *Δ*JLCA (*P* value)	Risk of JLCA decrease ≥ 2°: OR (95% CI, *P* value)	
		Univariable	Multivariable
*Demographic factors*			
Age	0.175 (0.121)	0.960 (0.889–1.036, 0.294)	
Sex (female)	0.136 (0.231)^a^	3.462 (0.416–28.819, 0.251)	
Height	− 0.012 (0.914)	1.004 (0.938–1.074, 0.912)	
Weight	− 0.136 (0.230)	1.022 (0.979–1.067, 0.324)	
BMI	− 0.192 (0.089)	1.107 (0.959–1.276, 0.164)	
*Radiographic parameters*			
Preoperative mFTA	**0.497 (< 0.001)**	**0.651 (0.525–0.809, < 0.001)**	**–**
Preoperative mLDFA	− 0.179 (0.113)	1.209 (0.913–1.600, 0.185)	
Preoperative mMPTA	0.136 (0.228)	0.765 (0.571–1.024, 0.072)	
Preoperative JLCA (standing)	**− 0.724 (< 0.001)**	**7.181 (2.711–19.021, < 0.001)**	**4.429 (1.350–14.534, 0.014)**
Preoperative JLCA (supine)	**− 0.227 (0.043**)	**1.718 (1.006–2.932, 0.047)**	**–**
Difference of JLCA standing and supine	**− 0.755 (< 0.001)**	**22.679 (4.415–116.490, < 0.001)**	**11.74 (1.770–77.886, 0.011)**
Preoperative TPS	− 0.083 (0.465)	1.110 (0.926–1.332, 0.260)	
Postoperative mFTA	0.018 (0.875)	0.896 (0.728–1.102, 0.299)	
Postoperative mMPTA	**− 0.232 (0.039)**	1.178 (0.973–1.426, 0.093)	
Postoperative JLCA (standing)	0.097 (0.394)	1.340 (0.805–2.230, 0.260)	
Postoperative JLCA (supine)	− 0.053 (0.642)	1.178 (0.711–1.954, 0.525)	
Postoperative TPS	− 0.121 (0.286)	1.127 (0.946–1.343, 0.180)	
Change of mFTA	**− 0.457 (< 0.001)**	**1.360 (1.135–1.629, 0.001)**	**–**
Change of mMPTA	**− 0.298 (0.007)**	**1.282 (1.062–1.547, 0.010)**	**–**
Change of TPS	− 0.049 (0.669)	1.032 (0.860–1.240, 0.733)	

*JLCA* joint-line convergence angle, *ΔJLCA* the amount of change of the JLCA after high tibial osteotomy, *OR* odds ratio, *CI* confidence interval, *BMI* body mass index, *mFTA* mechanical femorotibial angle, *mLDFA* mechanical lateral distal femoral angle, *mPTA* mechanical proximal tibial angle; *TPS* tibial posterior slope

^a^Spearman’s correlation analysis

JLCA change was best predicted by an equation in the multivariable linear regression model:

Change of JLCA = 0.478–0.812 x Preoperative JLCA (standing) + 0.514 x Preoperative JLCA (supine) [*R*^2^ = 0.640, *P* < 0.001].

The best prediction model for postoperative JLCA was:

Postoperative JLCA = 0.478 + 0.188 x Preoperative JLCA (standing) + 0.514 x Preoperative JLCA (supine) [*R*^2^ = 0.531, *P* < 0.001].

The preoperative JLCA in the supine position showed a better correlation and agreement with the postoperative JLCA than the preoperative JLCA in the standing position (correlation coefficient 0.700, *P* < 0.001, ICC = 0.823) with smaller mean difference (− 0.2° ± 0.9°, *P* = 0.021) (Table 4).

**Table 4:** Relation of preoperative joint-line convergence angle (JLCA) parameters with postoperative JLCA

Preoperative parameters	Mean difference ***(P*** value)	Correlation coefficient (***P*** value)	ICC (***P*** value)
Preoperative JLCA (standing)	0.9° ± 1.2° (< 0.001)	0.617 (< 0.001)	0.732 (< 0.001)
Preoperative JLCA (supine)	**− 0.2° ± 0.9°** (0.021)	**0.700** (< 0.001)	**0.823** (< 0.001)

*JLCA* joint-line convergence angle, *ICC* intraclass correlation coefficient

## Discussion

The most important finding of this study is that the JLCA decreases after HTO, reflecting the soft-tissue correction, and the amount of its change is substantial in the patients with severe preoperative varus limb alignment, greater preoperative JLCA on standing or when supine, greater differences in preoperative JLCA on sanding and when supine, and a greater amount of alignment correction. In addition, greater preoperative JLCA on standing (cut-off value: 4°), and greater difference of JLCA on standing and when supine (cut-off value 1.7°) are risk factors for greater JLCA decrease ≥ 2°. We also proposed prediction formulae for the postoperative JLCA and its change after HTO, which uses the preoperative JLCA on standing and when supine. This information might be helpful in achieving accurate alignment correction and in avoiding unintended overcorrection after HTO.

We found that the JLCA decreases by a mean of 0.9° after HTO after surgery. Our findings are similar to studies that reported JLCA change after a medial opening-wedge HTO, but the amount of the change was rather small compared to the results of previous studies (Table 5) [13, 16–18]. Park et al. reported no significant change of JLCA after surgery, but the study was conducted in patients who underwent closing-wedge HTO, which may affect the results [27]. Lee et al. found that the amount of limb alignment correction was correlated with greater JLCA change [16]. Our study population presented with less severe preoperative varus deformity and a smaller amount of alignment correction than other studies. This might influence the smaller change in JLCA. As previous studies reported a mean change of JLCA < 2°, we regarded the decrease of JLCA ≥ 2° as the meaningful change. Considering that the acceptable range of the HTO is narrow, mFTA 0–6° [1, 2, 26, 28], and beyond this range is frequently regarded in the literature as an outlier, the change of JLCA of 2° might affect the accurate correction after HTO. Lee et al. reported a correlation of greater JLCA change with overcorrection [16]. We found that 20% of the patients showed a decrease of the JLCA of ≥ 2°. These patients theoretically were at risk of unintended overcorrection if the effect of the soft-tissue correction was not considered. Therefore, the possibility of significant JLCA change should be considered during the preoperative planning and intraoperative procedure to achieve satisfactory alignment correction [8, 11, 19–21].

**Table 5:** Recent studies reported the change of the joint-line convergence angle (JLCA) after high tibial osteotomy

Author (year)	N (M/F)	Age (years)	BMI (kg/m^**2**^)	F/U	Mechanical femorotibial angle (mFTA)			Joint-line convergence angle		
					Preoperative	Postoperative	ΔmFTA	Preoperative	Postoperative	ΔJLCA
Oh (2016) [17]	69 (13/56)	54.4 ± 7.2	27.0 ± 3.2	6 W to 3 M	− 6.0° ± 4.0° (− 22.2 to − 0.3°)	3.3° ± 3.3° (4.9–10.6°)	9.4° ± 4.7° (1.4–24.5°)	1.8° ± 1.8° (− 3.1–9.8°)	0.5° ± 1.7° (− 5.3–5.9°)	− 1.2° ± 1.6° (− 7.0–2.0°)
Lee (2016) [16]	86 (20/66)	57 (41–72)	25.7	6 M	− 8.0° ± 3.9° (− 1 to − 17°)	3.4° ± 2.3° (− 2 to − 7°)	9.78°	3.4° ± 2.3° (− 1.5–10.4°)	2.1° ± 2.3° (7–2°)	− 1.3° (− 2.1–8.4°)
Shin (2016) [18]	50 (21/29); 47 (27/20)	63.9 ± 4.8; 63.5 ± 7.7	26.2 ± 3.2; 26.0 ± 2.6	Last F/U (≥ 6 W)	− 7.5° ± 3.1°; − 7.4° ± 4.2°	2.8° ± 1.8°; 2.5° ± 2.2°	10.3° ± 3.5°;9.9° ± 5.3°	3.6° ± 2.4°; 3.6° ± 1.6°	1.8° ± 2.0°; 1.9° ± 1.8°	− 1.8° ± 1.6°; − 1.7° ± 1.3°
Ogawa (2016) [13]	50 (22/28)	62.3 ± 7.4	N/A	6 M	− 9.6° ± 4.0°	3.2° ± 2.3°	12.8° ± 4.3°	4.6° ± 2.2°	2.7° ± 1.6°	− 2.0° ± 1.5°
Park (2017) [27]	100 (5/89)	58.7 ± 7.4 (41–72)	25.0 ± 2.7	3/6/12/24 M/last F/U	− 8.1° ± 2.9°	1.6° ± 1.9°	9.7° ± 3.0°	4.4°	3.9°; 4.0°; 4.1°; 4.2°; 4.3°	− 0.2° to − 0.5°
**The current study**	80 (13/67)	57.4 ± 7.4	26.3 ± 3.7	≥ 3 M	− 5.6° ± 3.4°	3.3° ± 2.7°	8.9° ± 3.5°	2.7° ± 1.6°	1.8° ± 1.1°	− 0.9° ± 1.2°

*BMI* body mass index, F/U follow-up when the postoperative. whole-leg radiographs were taken, *ΔmFTA* the amount of change in the mFTA after high tibial osteotomy, *ΔJLCA* the amount of change of the JLCA after high tibial osteotomy, *N/A* not applicable

As a large JLCA change can cause unintended overcorrection in coronal alignment after HTO [16], the factors affecting a large JLCA change should be investigated. Among various radiographic parameters, a large preoperative JLCA and greater difference in preoperative JLCA between the standing and supine positions were found to be the most important risk factors. To our best knowledge, the difference between the two JLCA values on standing and in the supine position was first reported in the literature as a risk factor for a large JLCA change after HTO. Ogata et al. reported that the postoperative JLCA on standing is similar to the preoperative JLCA in the supine position, and used supine radiography for the preoperative planning, which indirectly supports our findings [21]. Lee et al. reported that the magnitude of change of coronal limb alignment before and after HTO is correlated with the JLCA change [16]. Jang et al. compared the coronal limb alignment on the intraoperative fluoroscopy with the postoperative standing radiograph [19]. They reported that a large JLCA was associated with a discrepancy between the two measurements, suggesting that a large preoperative JLCA can be a source of correction error after HTO [19]. Sabharwal et al. also reported that a JLCA > 3° was associated with a discrepancy of mechanical axis deviation on supine fluoroscopy and a standing full-length radiograph [20]. We found that a preoperative JLCA ≥ 4° or the difference of preoperative JLCA on standing and in the supine position ≥ 1.7° was the cut-off value to predict a large JLCA decrease ≥ 2° after HTO in the ROC curve analysis. We recommend that clinicians treating patients with a large preoperative JLCA or a great difference in JLCA between the standing and supine positions should be cautious in preoperative planning for HTO and during the operation to avoid unintended alignment overcorrection.

We presented formulae that predict the postoperative JLCA and the amount of JLCA change derived from the multivariable linear regression model using preoperative radiographic parameters and demographic factors. However, the *R*^2^ values, the proportion in the formula that explains the overall variation, were only 0.531 and 0.695, respectively. Our formula can be helpful in predicting the effect of the JLCA change, but individual variation should be noted and further validation in different study population should be required. Dugdale et al. suggested a formula composed of the tibial plateau width and the difference in the lateral joint space opening between both knees, which can be used to calculate the angle caused by the pathological lateral joint-space opening [8]. They recommended subtracting the calculated angle from the planned correction angle based on the preoperative varus deformity [8]. However, their prediction method is not validated sufficiently in the literature, and its application is limited when a patient has bilateral pathology. Ogata et al. reported that the postoperative JLCA on a standing radiograph is similar to that measured on the preoperative supine radiograph [21]. Based on this finding, they recommended using a supine radiograph in preoperative planning [21]. We also found that the postoperative JLCA showed the best correlation and agreement with the preoperative JLCA on the supine radiograph among the preoperative JLCA measurements, with least mean difference (Table 4). Varus deformity is generally more severe in the standing position than in the supine in osteoarthritic knees, which is explained by the adduction moment on the medial side in the standing position [29, 30]. After HTO, the valgus overcorrection of the proximal tibia reduced the adduction moment of the knee, which might decrease the JLCA. The supine position without weight-bearing seems to be a similar condition to the post-HTO status with decreased adduction moment [30]. We are not sure that the supine, whole-leg radiograph is more suitable for preoperative planning than a standard standing radiograph, as we did not used the whole-leg, supine radiograph to measure the JLCA in the supine position. Rather, we believe that the supine radiograph can be helpful to predict whether the JLCA will decrease substantially after surgery, especially in patients with a greater preoperative JLCA in the standing position. The usefulness of a supine radiograph was also supported by Dugdale et al., in patients with lateral-ligament laxity [8].

Despite the theoretical risk of unintended overcorrection owing to the change of JLCA, there was no difference in the incidence of coronal alignment outliers between group I (JLCA change < 2°) and II (JLCA decrease ≥ 2°). We believe that our intraoperative technique might have affected this result, which can simulate the weight-bearing effect by applying the axial compression force to the hindfoot [14]. Kim et al. reported that they could achieve more accurate alignment correction by applying a valgus force intraoperatively under fluoroscopy when deciding the amount of correction [22]. Although the magnitude of the axial or valgus force might be variable to the operator, these intraoperative techniques can be realistic options in taking account of the effect of the soft-tissue correction.

This study has several limitations to be considered. First, it was a retrospective radiographic study. However, we performed a standard protocol at the same time point after surgery and took radiographs on the post-surgical outpatient visit. So, the study design may have minimal effects on the results of this radiographic study. Second, the radiographic measurements can be inherently influenced by the validity of the radiograph taken. As flexion and rotation of the knee joint can raise measurement error, we tried to obtain a standardized patient position during taking the radiograph with the patella facing forwards and in maximum extension [31, 32]. Third, the preoperative supine JLCA was measured on the short-knee radiograph, not on the whole-leg radiograph in the supine position, which was not available in this series. Fourth, the effect of the greater JLCA change on the functional outcome was not investigated. There was no difference in the final alignment correction accuracy between groups I and II; this may be due to our intraoperative axial compression technique. As the alignment accuracy is comparable, the clinical outcome is expected to be similar in our series, although the amount of JLCA change was different. A further long-term study is warranted.

## Conclusion

Change in JLCA can affect postoperative alignment after HTO. Surgeons should consider the effect of the JLCA change during the preoperative planning and intraoperative procedure of HTO to avoid unintended overcorrection, especially in patients with a preoperative JLCA ≥ 4° or the difference of preoperative JLCA in the standing and supine positions ≥ 1.7°. Information about JLCA changes can be helpful to estimate the effect of soft-tissue correction during HTO, but substantial individual variations should be considered.

## Data Availability

The datasets generated and/or analyzed during the current study are not publicly available but are available from the corresponding author on reasonable request.

## Abbreviations

HTO: High tibial osteotomy
JLCA: Joint-line convergence angle
OA: Osteoarthritis
SD: Standard deviation
BMI: Body mass index
AP: Anteroposterior
mFTA: Mechanical femorotibial angle
mMPTA: Mechanical medial proximal tibial angle
mLDFA: Mechanical lateral distal femoral angle
PACS: Picture archiving communication system
ICC: Intra-class correlation coefficient
ROC: Receiver operating characteristic
OR: Odds ratio
